# Double trouble for prostate cancer: synergistic action of AR blockade and PARPi in non-HRR mutated patients

**DOI:** 10.3389/fonc.2023.1265812

**Published:** 2023-09-21

**Authors:** Alexander Giesen, Loïc Baekelandt, Wout Devlies, Gaëtan Devos, Herlinde Dumez, Wouter Everaerts, Frank Claessens, Steven Joniau

**Affiliations:** ^1^ Department of Urology, University Hospitals Leuven, Leuven, Belgium; ^2^ Departments of Cellular and Molecular Medicine and Clinical and Experimental Medicine, Catholic University Leuven (KU Leuven), Leuven, Belgium; ^3^ Department of Oncology, University Hospitals Leuven, Leuven, Belgium

**Keywords:** prostate cancer, HRR genes, synergistic effect (combined treatment), PARP inhibition (PARPi), androgen receptor signaling inhibitor

## Abstract

Prostate cancer (PCa) is the most common cancer in men worldwide. Despite better and more intensive treatment options in earlier disease stages, a large subset of patients still progress to metastatic castration-resistant PCa (mCRPC). Recently, poly-(ADP-ribose)-polymerase (PARP)-inhibitors have been introduced in this setting. The TALAPRO-2 and PROpel trials both showed a marked benefit of PARPi in combination with an androgen receptor signaling inhibitor (ARSI), compared with an ARSI alone in both the homologous recombination repair (HRR)-mutated, as well as in the HRR-non-mutated subgroup. In this review, we present a comprehensive overview of how maximal AR-blockade via an ARSI in combination with a PARPi has a synergistic effect at the molecular level, leading to synthetic lethality in both HRR-mutated and HRR-non-mutated PCa patients. PARP2 is known to be a cofactor of the AR complex, needed for decompacting the chromatin and start of transcription of AR target genes (including HRR genes). The inhibition of PARP thus reinforces the effect of an ARSI. The deep androgen deprivation caused by combining androgen deprivation therapy (ADT) with an ARSI, induces an HRR-like deficient state, often referred to as “BRCA-ness”. Further, PARPi will prevent the repair of single-strand DNA breaks, leading to the accumulation of DNA double-strand breaks (DSBs). Due to the induced HRR-deficient state, DSBs cannot be repaired, leading to apoptosis.

## Introduction

Prostate cancer (PCa) represents the most prevalent cancer and the fifth most frequent cause of cancer-related mortality among men worldwide (1). About 88-95% of patients present with non-metastatic disease at the time of diagnosis. Despite initial local disease management, many of these patients will experience biochemical recurrence (BCR) during follow-up. Although there is no radiographic evidence of metastases at time of diagnosis, many of these patients already have micro-metastases that will cause metastatic relapse later (2, 3). At the time of metastatic relapse, most patients require systemic treatment using androgen deprivation therapy (ADT) combined with an androgen receptor signaling inhibitor (ARSI) with or without six cycles of docetaxel. Although most patients do well with this combination, a proportion of them will become treatment resistant and develop metastatic castration-refractory PCa (mCRPC) (4). Patients with mCRPC currently have limited treatment options. These include ARSI, cytotoxic chemotherapy, Lu-PSMA and Radium-223 (depending on previous treatments, the clinical and radiographic presentation at mCRPC). Recently, poly-(ADP-ribose)-polymerase inhibition (PARPi) has been introduced as a novel agent in combination with an ARSI in this patient population. PARP inhibition has been successfully applied for several years in gynecological tumors (breast and ovarian cancer) in BRCA-mutated patients. The rationale behind the use of PARPi in PCa can be found in the fact that, among others, a somatic/germline homologous recombination repair (HRR) gene mutation (mainly in BRCA2) plays an important role in development and progression of the disease (5). In the past year, several large phase III trials have investigated the use of PARPi in combination with an ARSI in the mCRPC setting. All trials (PROpel, TALAPRO-2, and MAGNITUDE) started with both HRR-mutated and HRR-not-mutated patients included. However, in MAGNITUDE, there was a preselection based on HRR-mutation status, while in TALAPRO-2 and PROpel, an unselected (all-comer) population with a sub-stratification based on HRR-mutation status was included. All three studies showed a marked increase in radiographic progression-free survival (rPFS), time to first subsequent therapy or death (TFST), and a trend towards better overall survival (OS) with the use of PARPi in combination with an ARSI, compared to an ARSI alone in the HRR-mutated population, with the greatest benefit in the BRCA-mutated patients (6–8). MAGNITUDE (Niraparib and Abiraterone Acetate and Prednisone [AAP]) discontinued the non-HRR mutated arm prematurely due to ineffectiveness in the pre-planned futility analysis (6). Both TALAPRO-2 (Talazoparib and Enzalutamide) and PROpel (Olaparib and AAP) were conducted in a population of unknown HRR status and showed a benefit for rPFS in the entire population. Moreover, these benefits were observed in both the HRR-mutated and the non-HRR mutated patients (7, 8). These results clearly suggested that the combination of PARPi and ARSI could have a synergistic effect, creating a situation similar to that seen in BRCA-mutated patients (often referred to as “BRCA-ness”). This “BRCA-ness” makes a PCa cell vulnerable to PARPi (9–11). Recently, multiple researchers are focusing on combining results from the largest phase III trials with the combination of PARPi and an ARSI to determine the overall benefit in a mCRPC population. In these pooled results, PARPi is significantly better than placebo in an HRRm+ and HRRm- patient population. All these trials however mention the probability of a synergistic effect between PARPi and ARSI, but none of them explain this synergy in detail (12–14). In this review, we do not focus on describing previously mentioned studies in detail, but we thoroughly describe the effects of both maximal androgen deprivation and PARP inhibition on the prostate cancer cell and hypothesize how a potential synergistic effect of combining both treatments can be explained at the molecular level in non-HRR-mutated patients.

## Mechanism of action

The concept of using PARPi in men with prostate cancer is rapidly gaining popularity. Since two of the three large phase III trials showed a significantly better rPFS not only in the HRR-mutated, but also in the HRR-non-mutated population, many questions arise about the mechanism behind this possible synergistic effect.

### Influence of AR-inhibition on the prostate cancer cell

**Figure 1:**
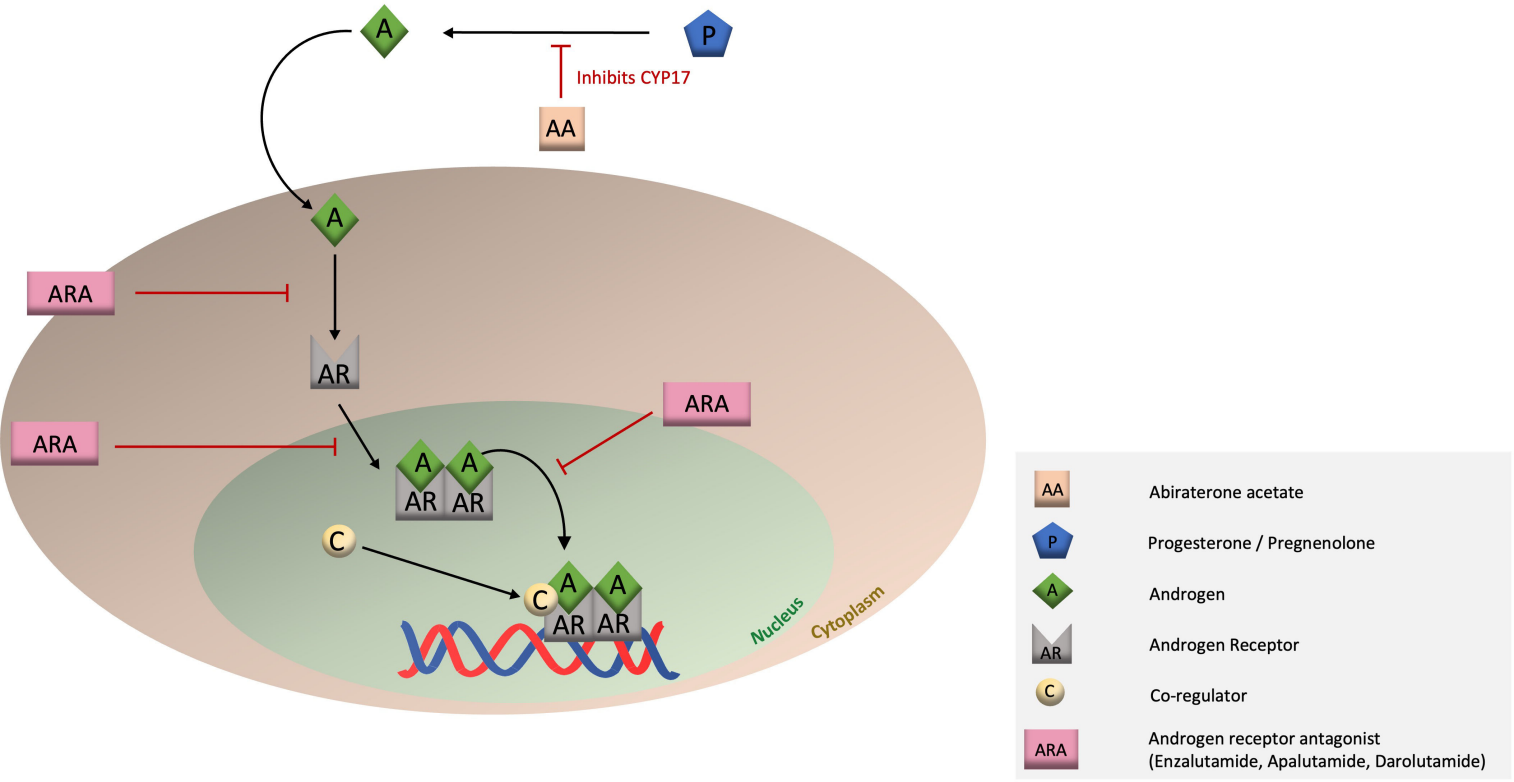
Mechanism of action of the different ARSIs.

Like prostate epithelial cells, prostate cancer (PCa) cells are primarily driven by stimulation of the androgen receptor (AR). AR stimulation by androgens provides survival- and growth-promoting signals for PCa cells (15). Androgen deprivation therapy (ADT), using LHRH agonists/antagonists or via orchiectomy, has for many years been the basis for the treatment of metastatic hormone-sensitive PCa (mHSPC). Eventually, all patients with mHSPC will develop evasive PCa cells that become resistant to AR blockade and thus become castration resistant (mCRPC) (16, 17). New potent drugs, second-generation antiandrogens, also called AR signaling inhibitors (ARSI), which can be an AR antagonist (enzalutamide, apalutamide or darolutamide) or an androgen synthesis inhibitor (abiraterone acetate (AA)), are currently available as a next-line treatment option to more deeply inhibit the AR at the molecular level (**Figure 1**). In fact, these drugs are now recommended as a first-line treatment, along with ADT, for mHSPC. These AR antagonists have a trifold mechanism of action. First, they competitively inhibit androgen binding to the AR (mainly dihydrotestosterone; testosterone to a lesser extent). Second, AR antagonists prevent translocation of the androgen-AR complex from the cytoplasm to the nucleus. Finally, the androgen-AR complexes in the nucleus are inhibited from dimerizing and binding to DNA and recruiting coactivators (e.g. PARP2), preventing transcription of downstream proliferation and survival pathways (18–22).

On the other hand, AA, the only clinically used second-generation androgen synthesis inhibitor, is an irreversible inhibitor of both 17,20-lyase and 17-alpha hydroxylase. Inhibition of CYP17 enzymes result in an inability to synthesize progesterone and pregnenolone into testosterone (23). Administration of AA results in a very strong inhibition of androgen production in the testis, adrenal glands and tumor cells, depriving the PCa of this stimulant. Due to the large suppression of androgen, transcription of downstream pathways of the AR is diminished as well (24).

The combination of ADT with an ARSI leads to increased suppression of the AR and decreased transcription of associated AR target genes. The most important of these associated effects is the emerging evidence of increased DNA damage and decreased DNA repair when treating PCa with an ARSI. The link between the AR and DNA damage repair (DDR) has been partially unraveled (11, 15, 25–31). In their study, Polkinghorn et al. showed that androgens could activate the transcription of several DDR genes (encoding DNA-PKcs, active in non-homologous end-joining (NHEJ), used in DNA double-strand break (DSB) repair, and PARP1, active in single-strand break (SSB) repair) in hormone-sensitive LNCaP cells (31). On the other hand, DNA damage can activate the AR, which in turn increases the expression of DDR genes such as DNA-PKcs proteins. Multiple preclinical studies have investigated this co-regulation between the AR and a DDR gene signature (29, 31–34). LNCaP, LNCaP-AR and VCaP cells treated with apalutamide or enzalutamide showed a significant increase in DNA damage even without radiation exposure (10, 31). Li et al. showed a significant increase in markers for DNA damage (γH2aX and RAD51), apoptosis and suppression of PCa growth after treatment of these cell lines with enzalutamide and Olaparib (compared to Olaparib alone) (10). Thus, by maximally suppressing the AR, the DDR pathway can be (partially) suppressed. When DSBs occur in these cells, they cannot be repaired, which will ultimately result in apoptosis. This artificial state of DDR-insufficiency induced by second-generation antiandrogen treatment is often referred to as ‘BRCA-ness’, as it produces an intracellular situation similar to that of a BRCA2-mutated patient when treated with PARP inhibition. However, the exact mechanism behind this ‘BRCA-ness’ is still not fully understood nor proven on human PCa tissue (9, 10, 35).

During the transition from mHSPC to mCRPC, there are numerous escape mechanisms that prostate cancer cells can develop to circumvent androgen or AR dependence. These escape mechanisms include, but are not limited to, AR splice variants, increased AR expression, and glucocorticoid receptor takeover (16, 17). Multiple genomic variations, including RB1 (a tumor suppressor gene) and BRCA2, have been associated with rapid resistance to second-generation antiandrogens, and thus progression to mCRPC (36–39). The frequency of HRR mutations has been shown to increase in advanced PCa (5, 40). Chakraborty et al. demonstrated that co-loss of BRCA2-RB1 (these genes are close together on chromosome 13q) induces invasiveness and a more aggressive PCa subtype. Adding PARPi to this BRCA2-RB1 co-loss showed promising results in reducing PCa growth (41).

### Influence of PARP-inhibition on the prostate cancer cell

Depending on the presence of single-strand breaks (SSBs) or double-strand breaks (DSBs), different DNA repair mechanisms are triggered. Each type of break initiates specific repair pathways, the most important of which are non-homologous end joining (NHEJ) and HRR for DSBs, and nucleotide excision repair (NER), base excision repair (BER) and mismatch repair (MMR) for SSBs (42). PARP1 is the most important and most researched nuclear enzyme of the PARP family. PARP1 is mostly involved in SSB DNA repair mechanisms in three ways: (i) detection of DNA damage, (ii) recruitment of repair factors, and (iii) regulation of biochemical activities (35, 43, 44). At the time of DNA damage, PARP1 contributes by detecting the location of SSBs, decompacting the chromatin structure and recruiting cofactors (e.g. XRCC1 and DDB2) to the damaged DNA region (43). PARPi’s are relatively new drugs in the PCa landscape; however, PARPi’s have been used much earlier in gynecologic malignancies (ovarian and breast cancer). PARPi’s bind PARP and lock the functional PARP to the DNA strand (“PARP trapping”), while inhibiting the execution of the other PARP functions. This stalls the replication fork, leading to the occurrence of DSBs (45).

**Figure 2:**
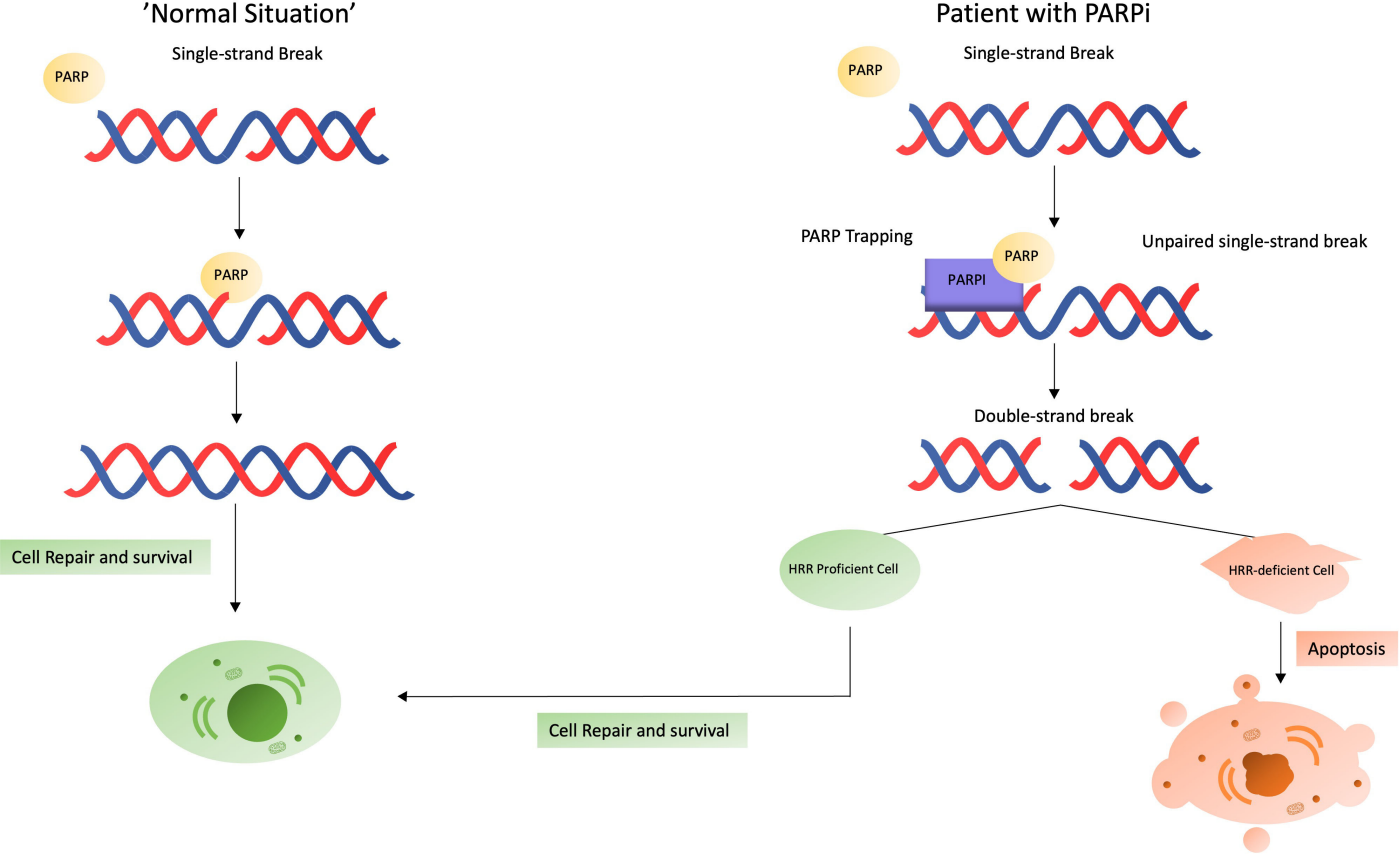
What happens in case of a single-strand break? Situation in a normal patient (left) versus in a patient receiving a PARPi (right).

Normally, the PARP1-initiated cascade by will repair the SSBs and restore the DNA strand without further problems. However, in the absence of functional PARP1 (e.g. PARP inhibition), there are two major consequences, representing the dual function PARP. First, when SSBs are encountered during DNA replication and cannot be repaired, as discussed above, the replication fork stalls and DSBs accumulate. DSBs in combination with a stalled replication fork are normally repaired via HRR. Even though NHEJ is an alternative option for DSB repair, in these situations, there seems to be an exclusion of NHEJ in favor of HRR (46). Thus, when the HRR pathway functions normally, DNA damage is repaired (normal cells). If the HRR pathway is dysfunctional (HRR-mutation, ‘BRCAness’ or BRCA-mutated patients), DSBs accumulate, leading to genomic instability until apoptosis occurs. Despite HRR being the favorable option, NHEJ will also come into action. However, in patients with PARP-inhibition, genomic instability is co-driven by NHEJ (47). Both scenarios are shown in **Figure 2**.

DSBs that accumulate and cannot be repaired before entering mitosis, drives the cancer cell in ‘mitotic catastrophe’, where these cell is prevented from proliferating and driven towards cell death or senescence (48). Hereby, the question regarding the effect of cells in a quiescence state arises. This quiescence state is known to be important for tumor cells to develop new evasive mechanisms through genomic changes (49, 50). The effect of PARPi in quiescent PCa cells is currently not known.

Second, in addition to its role in DNA damage repair, PARP1 is also involved in transcriptional regulation as a potent modulator of AR function and inducer of AR activity, and thus is involved in tumor proliferation. Schiewer et al. showed that PARP enzymatic activity is required for AR function in hormone-sensitive prostate cancer cells. Targeting PARP with PARP-inhibitors decreases AR activity and suppresses AR target gene expression, enhancing the activity of an ARSI. This is believed to prolong tumor doubling time and suppress the transition to CRPC. In patients with CRPC, PARP-inhibition plus castration significantly reduces tumor volume (30). PARP1 and PARP2 can also stimulate the AR to activate transcription of xenograft DNA repair genes (15, 30, 51).

Thus, PARPi exploits the dual function of PARP1 in DNA damage repair and AR regulation to suppress multiple pathways critical for prostate cancer cell survival and progression.

### Synergistic effect of the combination

As previously described, administration of a PARPi in a patient with a dysfunctional HRR pathway (e.g., BRCA2-mutation) will lead to accumulation of DSBs that cannot be repaired, leading to cell death. Drug-induced apoptosis, for example when the escape route is dysfunctional, is often referred to as ‘synthetic lethality’ (10, 52, 53). This phenomenon has been observed in all three phase III trials investigating PARPi in mCRPC. However, in TALAPRO-2 and PROpel, the combination of ARSI and PARPi also showed a significantly better rPFS compared to ARSI alone in the non-HRR-mutated patient population.

**Figure 3:**
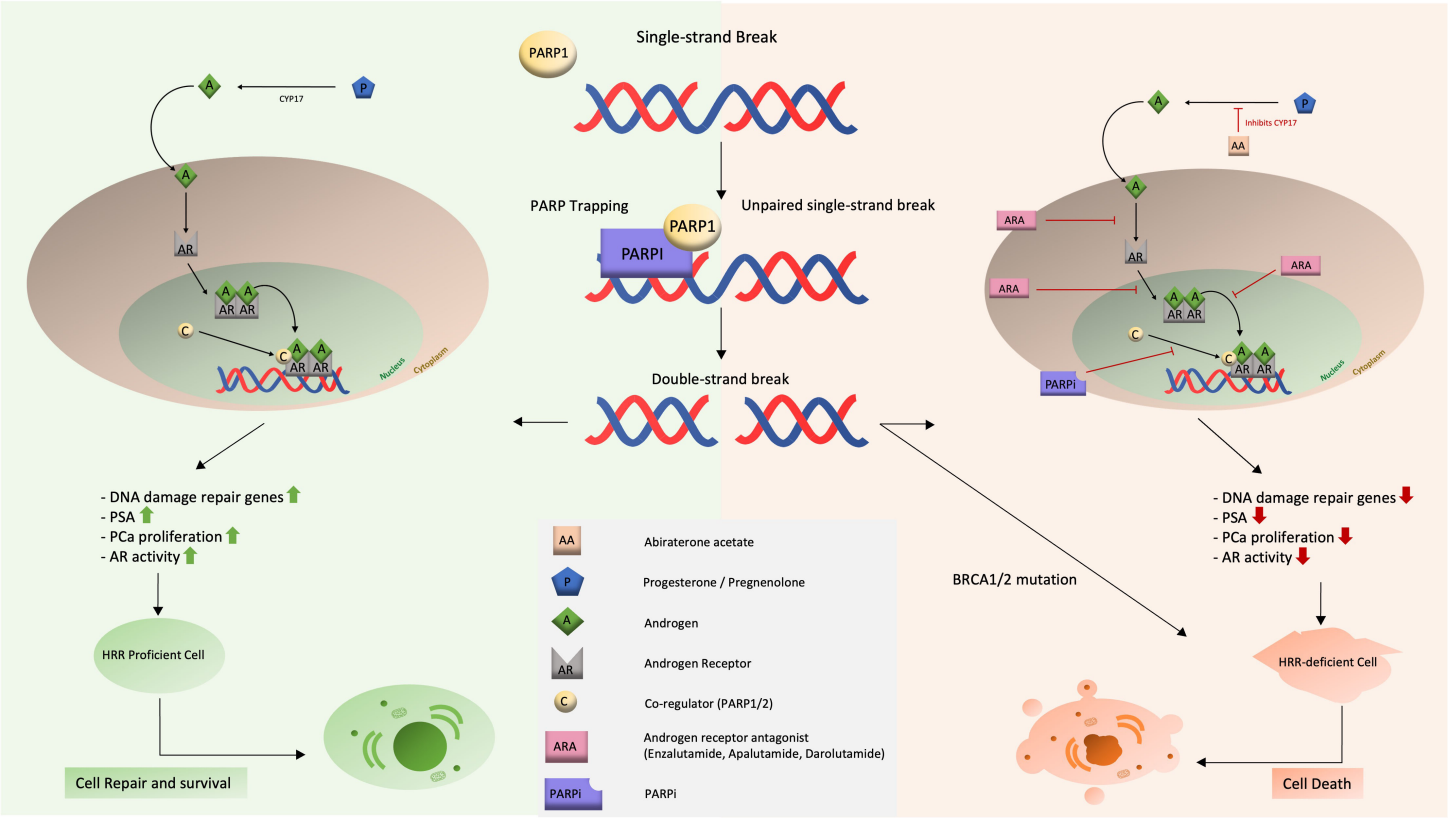
Schematic representation of the mechanism of action and synergistic effect of dual therapy with ARSI and PARPi.

This combination effect of ARSI and PARPi in non-HRR-mutated patients can be explained by a synergistic effect of both drugs (**Figure 3**). The combination of an ARSI and a PARP-inhibitor is hypothesized to act synergistically in two ways: (i) slowing or halting tumor growth and progression, (ii) while blocking DNA repair at all major pathways in PCa cells and thus ultimately inducing apoptosis.

We take a closer look at each synergistic effect.

First, the combination will slow or stop the growth and progression of the tumor on multiple levels. The ARSI blocks the AR deeply and at three levels (binding of an androgen, translocation and recruitment of cofactors), resulting in elimination of growth stimuli for the PCa. This lack of AR stimulation (and thus growth inhibitory effect) is enhanced by the addition of PARPi, which suppresses AR function and activity.

Second, DNA repair pathways are blocked, leading to cell death. This effect results directly from the strong inhibition of the AR. Since the AR activity is known to be a regulator of DNA repair genes, blockade of the AR will result in deficiency of the HRR pathway, inducing a ‘BRCAness’ situation. At this level, PARPi’s will do the job they are best known for: SSBs cannot be repaired, the replication fork stalls and DSBs accumulate, leading to apoptosis. It can be concluded that both agents reinforce each other’s mechanisms of action.

The therapeutic potential of PARPi’s has been tested in an explant of primary human tumors, showing that inhibition of the PARP1 enzyme leads to a decrease in tumor proliferation, all the more so when simultaneous inhibition of DSB repair via maximal androgen deprivation is achieved (28).

In addition, one of the escape mechanisms from AR-blockade using an ARSI, is deletion of the BRCA-gene (sometimes combined with co-loss of RB1). In this subgroup, the addition of a PARPi will act as in an HRR-mutated setting, leading to synthetic lethality (41).

Understanding the molecular basis behind this synergistic effect is crucial for future research in selecting optimal non-HRR-mutated patients for combination therapy with PARPi and an ARSI.

## Clinical evidence

On paper, this combination therapy looks promising. The phase III trials investigating PARPi in combination with ARSI for mCRPC have not yet been able to confirm or refute this synergistic effect. In MAGNITUDE (6), patients were preselected based on HRR-mutation status, while TALAPRO-2 (7) and PROpel (8) included an all-comer population and HRR-mutation status was determined after inclusion. These studies cannot be directly compared as they represent different study designs, gene testing strategies and previous exposure to an ARSI. These differences may cause biases. Well-designed prospective trials are needed to confirm this synergistic effect at the molecular level and its translation in favorable oncological outcomes in patients without an HRR-mutation. A neoadjuvant trial would suit this question best, since this design rules out previous effects of androgen deprivation therapy on the molecular structure of PCa.

The question arises whether this combination therapy offers sufficient synergism to also benefit HRR-mutated patients, or whether sequencing these drugs in mCRPC patients is a valuable option (since no “BRCA-ness” needs to be created). A phase II trial, BRCAAWAY (NCT03012321) is currently investigating this question. In BRCAAWAY, eligible mCRPC patients with BRCA1, BRCA2 or ATM mutation were randomized in one of three arms: (i) AAP, (ii) Olaparib, (iii) AAP + Olaparib with PFS as primary endpoint. Crossover at progression was considered in the first two arms. While final results have not yet been published, an interim analysis presented at ASCO 2022, showed a strong benefit of the combination therapy over either agent alone (54).

With the great results of using PARP-inhibition in a mCRPC setting, multiple new trials testing these PARP-inhibitors in a mHSPC population are starting. Currently, we are also waiting results of rucaparib together with enzalumatide in mCRPC patients (CASPAR trial).

An overview of previous and registered studies using a combination of PARPi and an ARSI can be found in **Table 1**.

**Table 1:** Overview of clinical trials with PARPi and ARSI in mCRPC and mHSPC.

Trials of PARPi in combination with an ARSI in mCRPC				
Clinical trial (NCT) + Phase	PARPi combination investigated	HRR selection (HRRm rate)	Primary Outcome	Reference
NCT01972217 – Phase 2	Olaparib + AA	Unselected (HRRm: 15% vs 14%)	Median rPFS 13.8 mo vs 8.2 mo (HR 0.65)	(55)
PROpel (NCT03732820) – Phase 3	Olaparib + AA	Unselected (HRRm: 27.8% vs 29%)	Median rPFS 24.8 vs 16.6 mo (HR 0.66)	(8)
MAGNITUDE (NCT03748641) – Phase 3	Niraparib + AA	A: HRR- (HRRm: 0%) B: HRR+ (HRRm: 100%)	A: Median rPFS not improved (HR 1.09) B: Median rPFS 16.5 mo vs 13.7 mo (HR 0.73)	(6)
NCI 9102 (NCT01576172) – Phase 2	Veliparib + AA	Unselected based on ETS (ETS fusion: 35.4% vs 33.8%)	PSA_50_ 72.4% vs 63.9% (*p = 0.27*)	(56)
TALAPRO-2 (NCT03395197) – Phase 3	Talazoparib + enzalutamide	Unselected (HRRm: 21% vs 21%)	Median rPFS ‘Not reached’ vs 21.9 mo (HR 0.63)	(7)
CASPAR (NCT04455750) – Phase 3	Rucaparib + enzalutamide	Unselected	rPFS and OS	/
BRCAAway (NCT03012321) – Phase 2	Triple design with possibility of cross-over: olaparib + AA vs olaparib vs AA)	HRR+	PFS	/
**Trials of PARPi in combination with an ARSI in mHSPC**				
TALAPRO-3 (NCT04821622) – Phase 3	Talazoparib + enzalutamide	DDR+	rPFS	/
NCT04734730 – Phase 2	Talazoparib + AA (single arm)	Unselected	PSA nadir <0.2 ng/ml	/
ZZ-first (NCT04332744) – Phase 2	Talazoparib + enzalutamide	Unselected	PSA complete response	/
AMPLITUDE (NCT04497844) – Phase 3	Niraparib + AA	HRR+	rPFS	/
NCT05167175 – Phase 2	Olaparib + AA (single arm)	HRR+	rPFS	/

Published trials (except NCI 9102 (56)) used patients HRR-mutation status for randomization and/or subanalysis to determine PARP-inhibition efficacy in mCRPC patients. However, previous studies found other important mutations associated with DSB occurrence and PARP-inhibition efficacy. Gene fusions are present in around 50% of PCa (57). The most prevalent of these molecular aberrations are mainly gene fusions of TMPRSS2 (an androgen-regulated gene) together with E-twenty six (ETS) family members (ERG and ETV1). This fusion gene can lead to an increase in DSBs via inhibiting NHEJ though decreased expression and activity of DNA-PKcs, thus also resulting in synthetic lethality when administering PARP-inhibition (57, 58). These molecular aberrations could potentially partially explain the beneficial effect in HRR-non-mutated patients. Despite the negative results of the NCI 9102 trial (56) regarding PSA_50_, investigation around these aberrations in clinical trials can be indicated.

## Conclusions

New treatment modalities are emerging for patients with metastatic castration-resistant prostate cancer (mCRPC). Recently, poly-(ADP-ribose)-polymerase (PARP)-inhibition has been introduced in mCRPC. Phase III trials (MAGNITUDE, TALAPRO-2 and PROpel) showed a clear benefit in radiographic progression-free survival (rPFS), time to first subsequent therapy or death (TFST), and a trend towards better overall survival (OS) in HRR-mutated patients. However, TALAPRO-2 and PROpel also demonstrated this benefit in rPFS in a non-HRR-mutated patient population. While lacking real-world confirmation, preclinical studies showed that maximal inhibition of the androgen receptor (AR) via an AR signaling inhibitor (ARSI) resulted in arresting tumor growth and progression while downregulating HRR gene expression. This results in a “BRCA-ness” situation, which can be exploited with concomitant PARPi. Well-designed proof-of-concept studies are needed to confirm this concept.

## Author contributions

AG: Conceptualization, Investigation, Methodology, Writing- original draft, Writing- review & editing. LB: Visualization, Writing- original draft. WD: Conceptualization, Data curation, Investigation, Writing- original draft. GD: Writing- review & editing, Conceptualization. HD: Supervision, Writing- review & editing. WE: Supervision, Writing- review & editing. FC: Supervision, Writing- review & editing. SJ: Supervision, Writing- review & editing.
